# Molecular Measurable Residual Disease Testing of Blood During AML Cytotoxic Therapy for Early Prediction of Clinical Response

**DOI:** 10.3389/fonc.2018.00669

**Published:** 2019-01-15

**Authors:** Hong Yuen Wong, Anthony D. Sung, Katherine E. Lindblad, Sheenu Sheela, Gregory W. Roloff, David Rizzieri, Meghali Goswami, Matthew P. Mulé, Nestor R. Ramos, Jingrong Tang, Julie Thompson, Christin B. DeStefano, Kristi Romero, Laura W. Dillon, Dong-Yun Kim, Catherine Lai, Christopher S. Hourigan

**Affiliations:** ^1^Laboratory of Myeloid Malignancies, Hematology Branch, National Heart, Lung and Blood Institute, Bethesda, MD, United States; ^2^Duke University School of Medicine, Durham, NC, United States; ^3^Office of Biostatistics Research, Division of Cardiovascular Sciences, National Heart, Lung and Blood Institute, Bethesda, MD, United States

**Keywords:** acute myeloid leukemia (AML), MRD, remission, measurable residual disease (MRD), WT1 = Wilms tumor 1, somatic mutations in cancer, Next Gen Sequencing (NGS)

## Abstract

Measurable residual disease (MRD) testing after initial chemotherapy treatment can predict relapse and survival in acute myeloid leukemia (AML). However, it has not been established if repeat molecular or genetic testing during chemotherapy can offer information regarding the chemotherapy sensitivity of the leukemic clone. Blood from 45 adult AML patients at day 1 and 4 of induction (*n* = 35) or salvage (*n* = 10) cytotoxic chemotherapy was collected for both quantitative real-time PCR (qPCR) assessment (*WT1*) and next generation sequencing (>500 × depth) of 49 gene regions recurrently mutated in MDS/AML. The median age of subjects was 62 (23–78); 42% achieved a complete response. *WT1* was overexpressed in most patients tested but was uninformative for very early MRD assessment. A median of 4 non-synonymous variants (range 0–7) were detected by DNA sequencing of blood on day 1 of therapy [median variant allele frequency (VAF): 29%]. Only two patients had no variants detectable. All mutations remained detectable in blood on day 4 of intensive chemotherapy and remarkably the ratio of mutated to wild-type sequence was often maintained. This phenomenon was not limited to variants in *DNMT3A, TET2*, and *ASXL1*. The kinetics of *NPM1* and *TP53* variant burden early during chemotherapy appeared to be exceptions and exhibited consistent trends in this cohort. In summary, molecular testing of blood on day 4 of chemotherapy is not predictive of clinical response to cytotoxic induction therapy in AML. The observed stability in variant allele frequency suggests that cytotoxic therapy may have a limited therapeutic index for clones circulating in blood containing these mutations. Further validation is required to confirm the utility of monitoring *NPM1* and *TP53* kinetics in blood during cytotoxic therapy.

## Introduction

The use of high sensitivity techniques to measure residual leukemic burden in patients achieving a complete remission by cytomorphological criteria is increasingly considered part of the standard of care for acute myeloid leukemia (AML) (1–4). While testing for measurable residual disease (MRD) in AML is typically performed using multi-parameter flow cytometry (MPFC) or real-time quantitative PCR (qPCR) there is increasing recent research interest in the potential of sequencing-based approaches(5–14). The results of MRD testing in AML appear prognostic when measured at key landmark timepoints following initial therapy, typically after 1–2 cycles of induction therapy or before allogeneic transplantation (15–21). It is currently not known if testing changes in residual leukemic burden at earlier timepoints, for example during initial induction therapy, would have clinical utility.

We used two independent molecular techniques for AML MRD quantification, *WT1* expression by qPCR and targeted DNA sequencing for common MDS/AML variants. The Wilms tumor gene *WT1* is expressed in approximately 90% of cases of AML and has been extensively tested and standardized as a method of MRD detection in AML (22, 23). The utility of *WT1* testing is limited to a subset of AML MRD cases and more recently the quantitative assessment by DNA sequencing of variants in genes known to be recurrently mutated in myeloid malignancies has been proposed as a more broadly applicable measure of AML MRD (6, 9, 14). We used both these molecular techniques to determine if early assessment of blood from AML patients during the first 4 days of intensive cytotoxic therapy can predict subsequent clinical response.

## Methods

### Patients and Sample Collection

**Figure 1:**
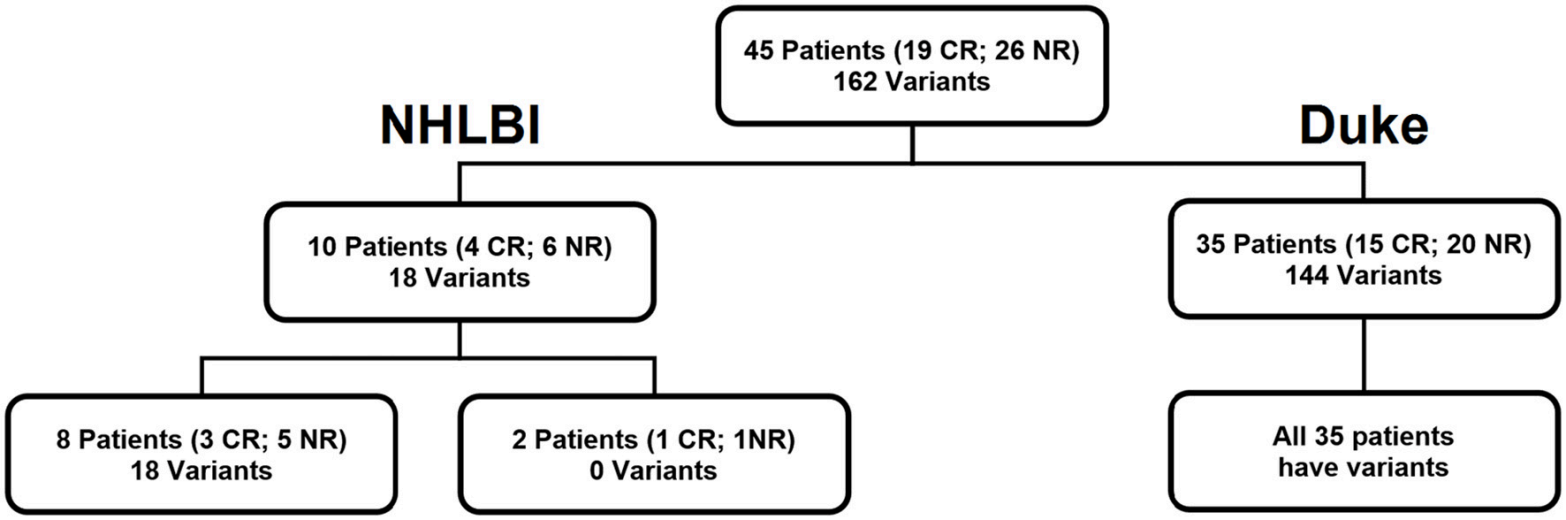
Cohort of 45 patients. Our patient cohort consisted of 10 patients who received salvage chemotherapy at NHLBI, NIH (NCT02527447) and 35 newly diagnosed patients who received induction chemotherapy at Duke University School of Medicine. Variants refers to the number of targets identified by DNA sequencing.

Blood was collected daily from day 1 through at least day 5 of intensive cytotoxic therapy from 45 adult AML patients with a median age of 62 years-old (range: 23–78) following informed consent on IRB-approved protocols (Figure 1 and Table 1, Tables S1, S2).

**Table 1:** Patient characteristics.

**Cohort**	**NHLBI(%)**	**Duke(%)**	**Total(%)**
Number of patients	10 (100)	35 (100)	45 (100)
Male	5 (50)	18 (51)	23 (51)
Female	5 (50)	17 (49)	22 (49)
Age median	52	63	62
Age range	23–66	30–78	23–78
**ELN RISK STRATIFICATION BY GENETICS**			
Favorable	1 (10)^*^	11 (31)	12 (27)
Intermediate	3 (30)^*^	10 (29)	13 (29)
Adverse	6 (60)^*^	9 (26)	15 (33)
Unclassified	0	5 (14)	5 (11)
**REMISSION STATUS AFTER INDUCTION**			
Clinical remission (CR)	4 (40)	15 (43)	19 (42)
Non-responder (NR)	6 (60)	20 (57)	26 (58)

* *Risk stratification on the basis of cytogenetics and molecular features in patients with refractory or relapsed AML may not be clinically informative and is shown here only for reader's interest*.

Ten patients (median age: 52, range: 23–66) had relapsed or refractory AML (RR-AML) and were recruited to the National Heart, Lung, and Blood Institute (NHLBI) at the National Institutes of Health to receive salvage chemotherapy (NCT02527447). Nine of these patients were treated with EMA (cytarabine 500 mg/m^2^ CI days 1–3 and days 8–10, mitoxanthrone 12 mg/m^2^ days 1–3, and etoposide 200 mg/m^2^ CI days 8–10) with the other receiving G-CLAC (G-CSF, clorafabine and high dose cytarabine) (Table S2).

Thirty-five patients (median age: 63, range: 30–78) were treated at Duke University School of Medicine with induction chemotherapy (“7+3,” 7 days of continuous infusion cytarabine with 3 days of anthracycline) for newly diagnosed AML.

In addition, research samples from clinically indicated bone marrow examinations and post-induction follow-up visits were available for a subset of 22 patients.

Blood was also collected from healthy adult donor subjects following informed consent on an IRB-approved protocol.

### Nucleic Acid Extraction From Blood and Marrow

Samples were processed with NucleoSpin Blood QuickPure kits (Machery-Nagel) and NucleoSpin RNA Blood kits (Machery-Nagel), as per manufacturer's instructions (NIH) or stored in PAXgene Blood/Marrow RNA tubes (PreAnalytiX) at Duke and shipped frozen to the NIH. Upon thawing PAXgene tubes, 2 mL for gDNA isolation were pelleted and resuspended in PBS then processed with the QIAamp DNA blood mini kit (QIAGEN), as per manufacturer's instructions. The remaining volume was processed with the PAXgene. Blood RNA Kit (PreAnalytiX) as per manufacturer's instructions (for yields see Figure S1).

### Real-Time Quantitative PCR (qPCR)

RNA (130–260 ng) was reverse transcribed using the RT^2^ First Strand kit (330404, QIAGEN). When necessary, RNA was concentrated with a Savant SVC-100H centrifugal evaporator. Resultant cDNA was loaded into a Custom RT^2^ Profiler PCR Array containing lyophilized qPCR primers for *WT1* and *ABL1* using the QIAgility System (QIAGEN). qPCR was performed (hold 2 m at 50°C, hold 10 m at 95°C, then 50 cycles of 15 s at 95°C and 60 s at 60°C) on the Rotor-Gene Q Platform (QIAGEN) and Ct values were collected with a threshold of 0.06. Healthy levels of *WT1* expression were established based on upper limit observed in blood of 34 healthy donors.

### DNA Sequencing Using a Myeloid Panel During Chemotherapy

A total of 49 gene or gene regions recurrently mutated in MDS and/or AML were sequenced using amplicon-based targeted DNA sequencing (RainDance, Billerica, MA). This included genes with functions in methylation, chromatin-cohesin, signaling, transcription as well as *TP53, NPM1*, and others (see Table S3). Libraries were prepared from 100 ng of gDNA and paired-end 300 bp sequencing was performed on the MiSeq instrument (Ilumina), as per manufacturer's instructions. Results were analyzed using the NextGENe v2.4.2.1 software (SoftGenetics, PA). Sequences were aligned to human genome build v37 (hg19). Non-coding and synonymous variants, along with known sequencing artifacts and regions with <500-fold coverage were removed. Remaining variants (i.e., missense, in-frame, frameshift, and non-sense mutations) with variant allele frequencies (VAFs) above 5% at either day 1 or 4 were considered in subsequent analyses.

### Custom DNA Sequencing for MRD Tracking After Chemotherapy

In order to track variants in longitudinal blood samples in 2 patients, a custom DNA sequencing assay was designed (DB0188, VariantPlex, ArcherDX). Libraries were prepared from 400 ng gDNA; using paired-end 150 bp sequencing on MiSeq instrument (Ilumina), as per manufacturer's instructions. Archer Analysis software version 5.1.3 was used for analysis.

### Statistical Analysis

All statistical analyses were performed using Prism v7.02 (GraphPad Software, CA). The Wilcoxson signed-rank test was performed on paired D1/D4 VAFs. Additive (D4–D1, with denominator of D1 or D4) and multiplicative (D4/D1) differences between D1 and D4 were calculated, then the Wilcoxson rank-sum test was performed on these differences between CR/NR groups. The Chi-square test was performed based on whether the additive difference was positive or negative between CR and NR groups. The unpaired *t*-test was performed on relative expression levels of *WT1*. For all statistical tests, *P* < 0.05 was considered significant.

## Results

### Patients and Sample Collection

Full demographics, risk classification, treatment and responses are listed in Table 1, Tables S1,S2. Overall, 19 of 45 patients achieved a complete remission (CR) after intensive therapy (42%). Average white blood cell count was 10 K/ul (range: 0.3–60) on day 1 decreasing to 2.3 K/ul (range: 0.2–26) by treatment day 4. Obtaining sufficient quantities of nucleic acid from blood is the limiting factor for molecular testing during these early time points of cytotoxic chemotherapy in AML. All 45 patients had sufficient DNA for sequencing but only 34/45 patients had enough RNA from paired day 1 and 4 samples for qPCR analysis. There was insufficient RNA and DNA yields from blood beyond day 4 in most patients (Figure S1).

### *WT1* Expression Level on Day 4 Is an Uninformative Biomarker of Clinical Response

**Figure 2:**
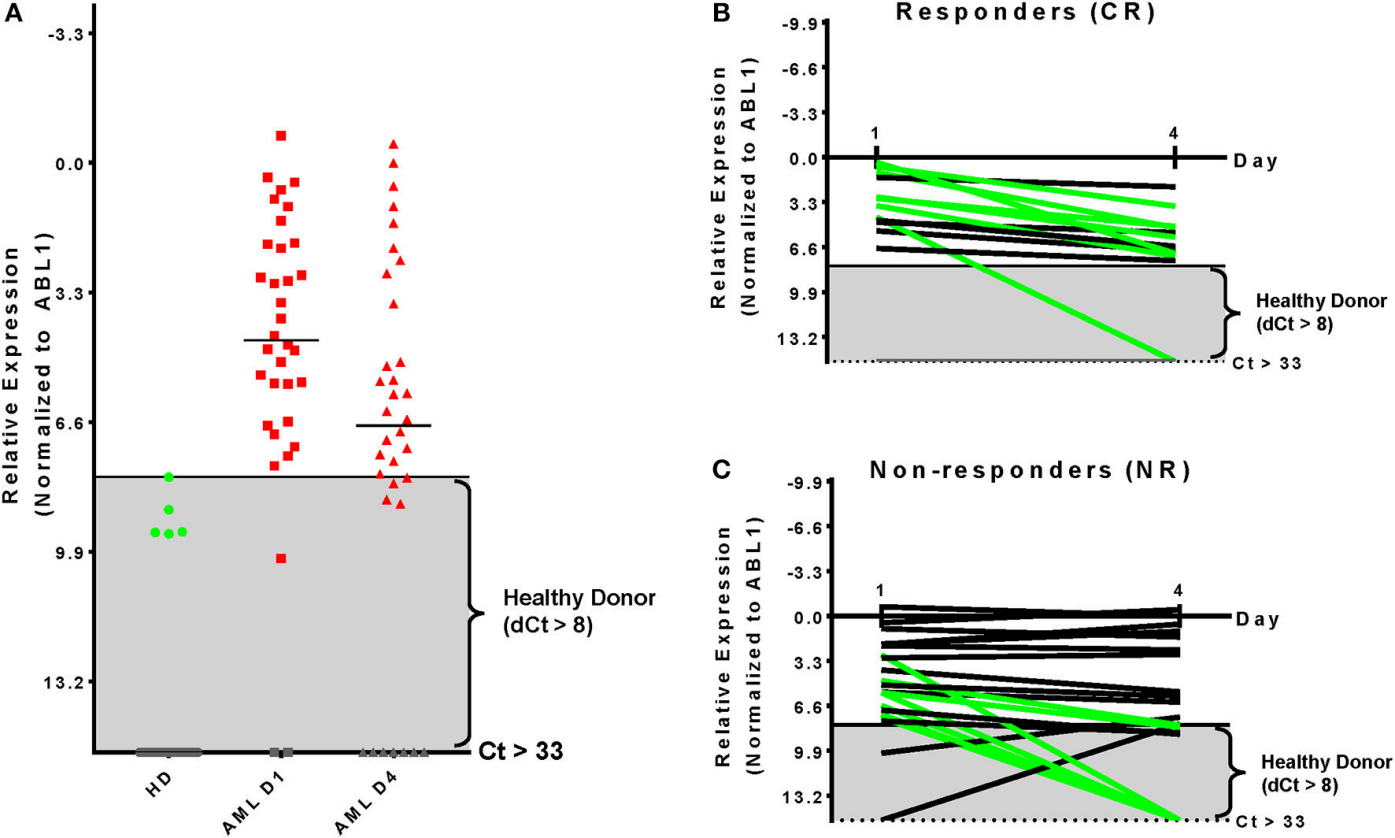
Kinetics of *WT1* expression during chemotherapy is uninformative for clinical response. **(A)** Thresholds for *WT1* overexpression in blood for this assay were established based on the upper limit observed by qPCR in 34 healthy donors and were consistent with previous reports (24). AML patient blood samples showed overexpressed *WT1* levels in 31 (91%) on Day 1 and 23 (68%) at Day 4. **(B)** Patients achieving a complete remission after therapy had at least a 4-fold reduction of *WT1* expression during therapy in 7 of 13 cases evaluated by qPCR (54%); 5/13 (38%) had <4-fold reduction; 1/13 (8%) had undetectable *WT1* levels. **(C)** Non-responding patients had at least a 4-fold reduction of *WT1* expression in 7 of 21 evaluated by qPCR (33%) NR; 14/21 (67%) had less than a 4-fold change. Two patients initially had *WT1* levels that were not overexpressed, but became so by treatment day 4. qPCR, quantitative real-time PCR; Green, at least 4-fold decrease; Black, <4-fold change; Gray, undetectable; Gray box indicates healthy donor range.

*WT1* gene expression (normalized to *ABL1* expression) was determined in 34 patients with sufficient RNA for qPCR analysis isolated from blood on days 1 and 4 of treatment. Compared with the upper limit of expression observed in healthy donors (Figure 2A) 31 of 34 patients overexpressed *WT1* on Day 1 (91%) consistent with prior reports (22, 23). By day 4 of induction therapy 23 patients had *WT1* over-expression. 7 of 13 (54%) patients achieving a CR had at least a 4-fold reduction in *WT1* expression, although notably 6 of these 7 patients remained overexpressed compared with healthy donors on day 4 (Figure 2B). Seven of 21 (33%) patients who were non-responders (NR) also had at least a 4-fold reduction in *WT1* expression. Two NR patients with undetectable *WT1* on day 1 had low level expression on day 4 (Figure 2C). Overall, changes in *WT1* expression in blood between day 1 and 4 of intensive cytotoxic chemotherapy for AML appear uninformative as a biomarker for clinical response in this cohort.

### DNA Sequencing for Variants Associated With Myeloid Malignancy Pre-treatment

**Figure 3:**
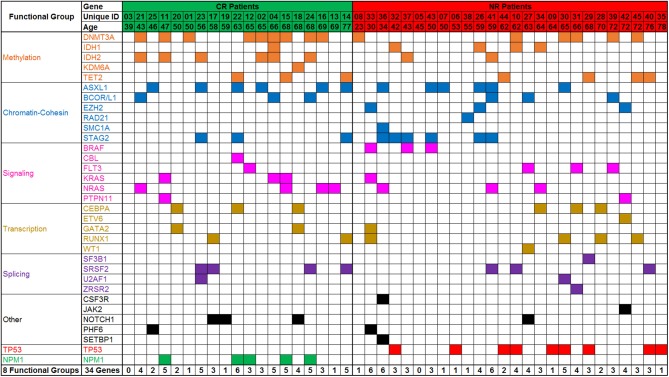
Targeted DNA sequencing of AML patient blood from day 1 of treatment. Variants were detected in 43 of 45 patients assessed. A total of 162 variants were identified in 34 genes or gene regions of 49 assessed. Genes with variants are grouped by gene function/class. Frequency of mutations and patterns of co-mutation are consistent with previous reports. CR, complete remission; NR, non-responder.

**Figure 4:**
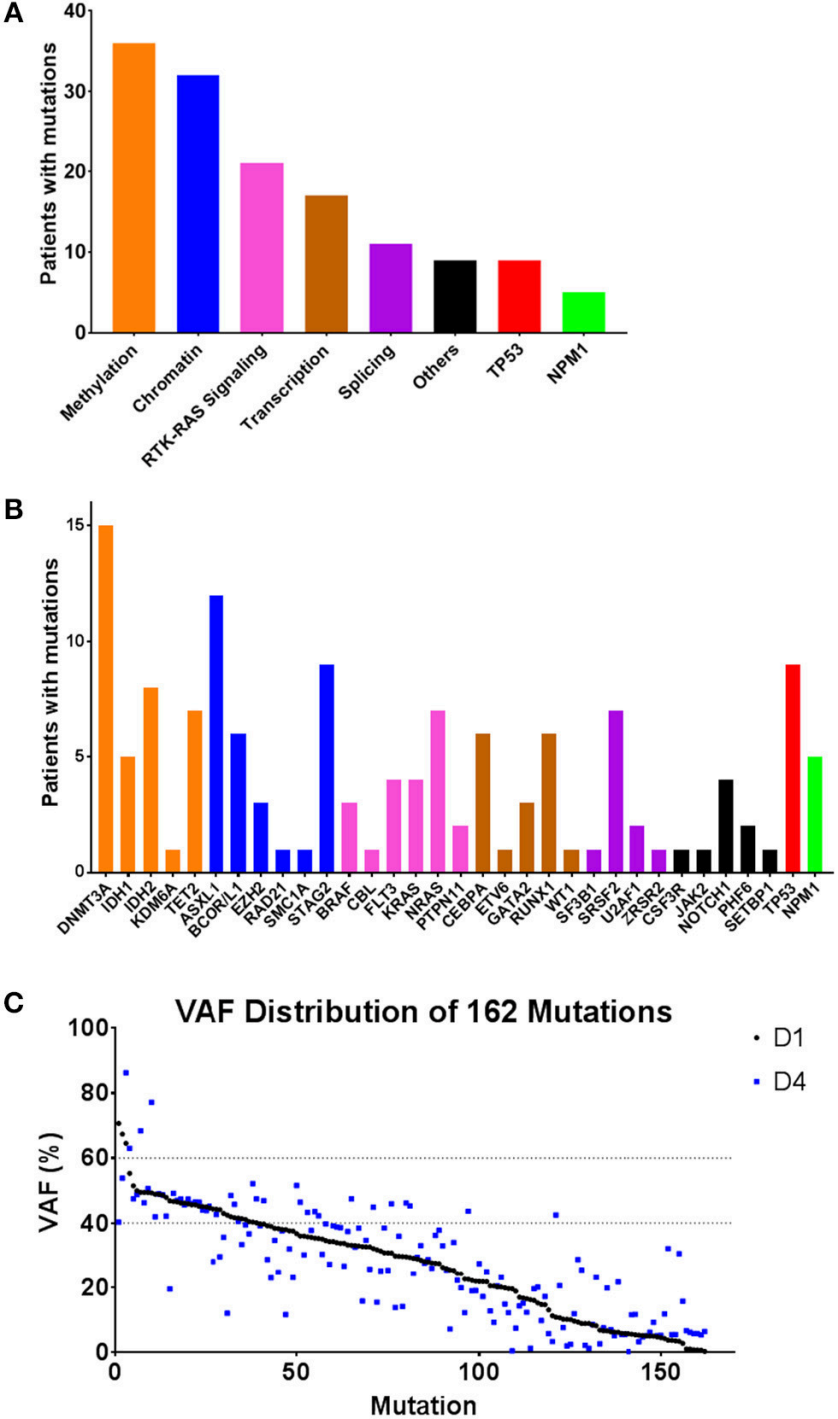
Summary of variants observed by DNA sequencing. **(A,B)** Frequency of variants summarized by gene function/class **(A)** and by individual gene name **(B)**. **(C)** Variant allele frequency (VAF) distribution of variants detected in 43 patients by targeted DNA sequencing, sorted by VAF on day 1 (black), with VAF of same variant in same patient from day 4 also shown (blue). Day 1 variants had a median VAF of 29.24% (range: 0.19–78.94%) whereas Day 4 variants had a median VAF of 27% (range:0.14–86.28%).

All patients had targeted DNA sequencing (Table S3) of blood taken on day 1 and 4 of chemotherapy. An average of 4 coding variants (range 0–7) were identified per patient, and only two patients had no variants suitable for disease tracking available (Figure 3). A total of 163 variants were found in 43 patients and some patients had multiple variants found within a single gene (considering multiple variants in one gene in the same patient as single event results in a total of 140 mutated genes). The most frequently mutated gene regions were consistent with those previously reported (25, 26) (see Figure 3, Figures 4A,B). There was no difference in the number of coding variants detectable at baseline in CR vs. NR patients (Figure 3). Variant allele frequencies (VAF) on day 1 were a median of 29% (range: 0.2–71%) with 75% having a VAF <40% (Figure 4C) on day 1. Consistent with current prognostic risk classifications (1), the *TP53* and *NPM1* mutation classes had predictive significance, with 9 of 9 patients with *TP53* mutations not achieving remission while all 5 of 5 patients with *NPM1* mutations achieved CR.

### Detectable Variant Allele Frequency Kinetics in Blood During Chemotherapy

Of the 43 patients with at least 1 variant detected in blood at day 1, 18 (42%) achieved CR and 25 (58%) were NR after induction therapy. Although white blood cell (WBC) count decreased 75% on average in the first 4 days of therapy Figure S1A), all variants detected in the blood on day 1 of chemotherapy remained detectable in blood on day 4.

**Figure 5:**
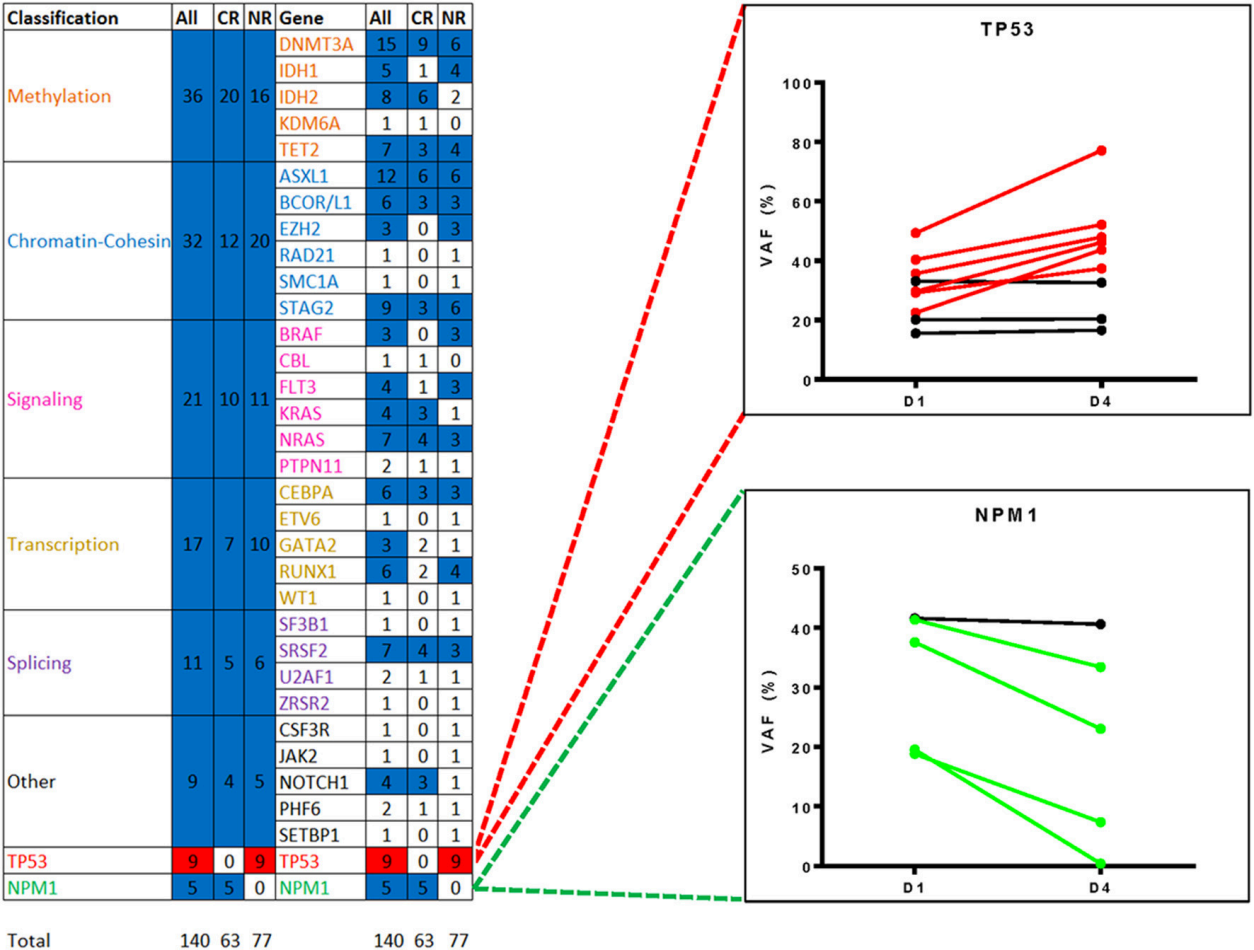
Changes in variant frequency during chemotherapy. All variants identified by targeted DNA sequencing in blood from day 1 of treatment (median VAF of 29%, range: 0.2–71%) were also detectable in blood from day 4 (median VAF 27%, range: 0.1–86%). Heatmap shows statistically significant changes in VAF between day 1 and 4 by either gene functional class or by individual gene/gene region, for all patients or just those with complete remission (CR) or non-responder (NR). Variants were only counted once per gene region per patients. Mutated *TP53*, detected only in NR patients, was significantly different (red, *P* < 0.05) and mutated *NPM1*, detected only in CR patients, demonstrated a consistent trend (blue, *P* = 0.0625). The remaining functional groups and individual genes were either non-significant (blue) or had too few data points for analysis (white). VAF, variant allele frequency.

**Figure 6:**
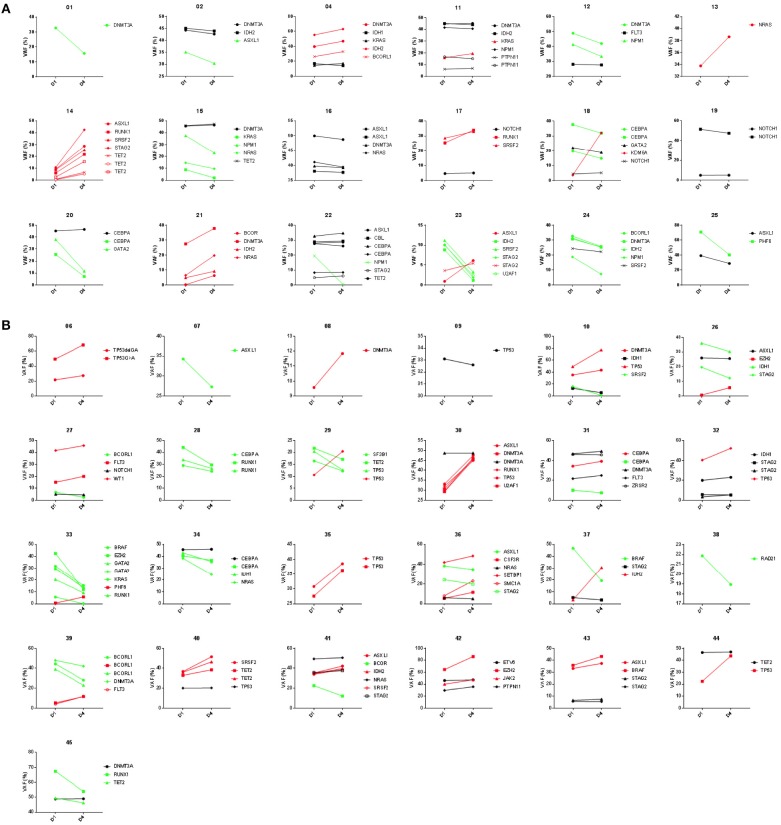
Variant frequency kinetics in blood from day 1 to 4 of AML chemotherapy. Results of targeted DNA sequencing on paired day 1 and 4 samples shown for 18 patients achieving complete remission **(A)** and 25 patients not responding **(B)** after induction chemotherapy. Trendlines highlight variants increasing (red) or decreasing (green) during induction therapy.

Variant allele frequencies (VAFs) on day 1 and 4 were compared based on the hypothesis that changes in detectable mutation burden in blood very early during intensive cytotoxic treatment may correlate with clinical response as later assessed by morphological examination of bone marrow at count recovery (i.e.,: approximately 30 days later). Day 1 and 4 VAFs, at both the genetic functional class level and individual gene level, were compared using the Wilcoxon Sign Test, for all patients and also for the subgroups achieving either CR or NR (Figures 5, 6). TP53 mutations were only detected in NR patients and showed a significant difference in VAF between days 1 and 4 (*P* < 0.05) with a mean increase of 34% (*n* = 9; range: −2 to 94%). NPM1 mutations were observed only in CR patients with a mean decrease of −44% (*n* = 5; range: −2 to −98%) (*P* = 0.0625). Furthermore, additional testing for 2-sample statistical significance of the additive and multiplicative differences was assessed between Day 1 and 4 VAFs at the genetic functional class level between the CR and NR patient groups, all of which were non-significant.

**Figure 7:**
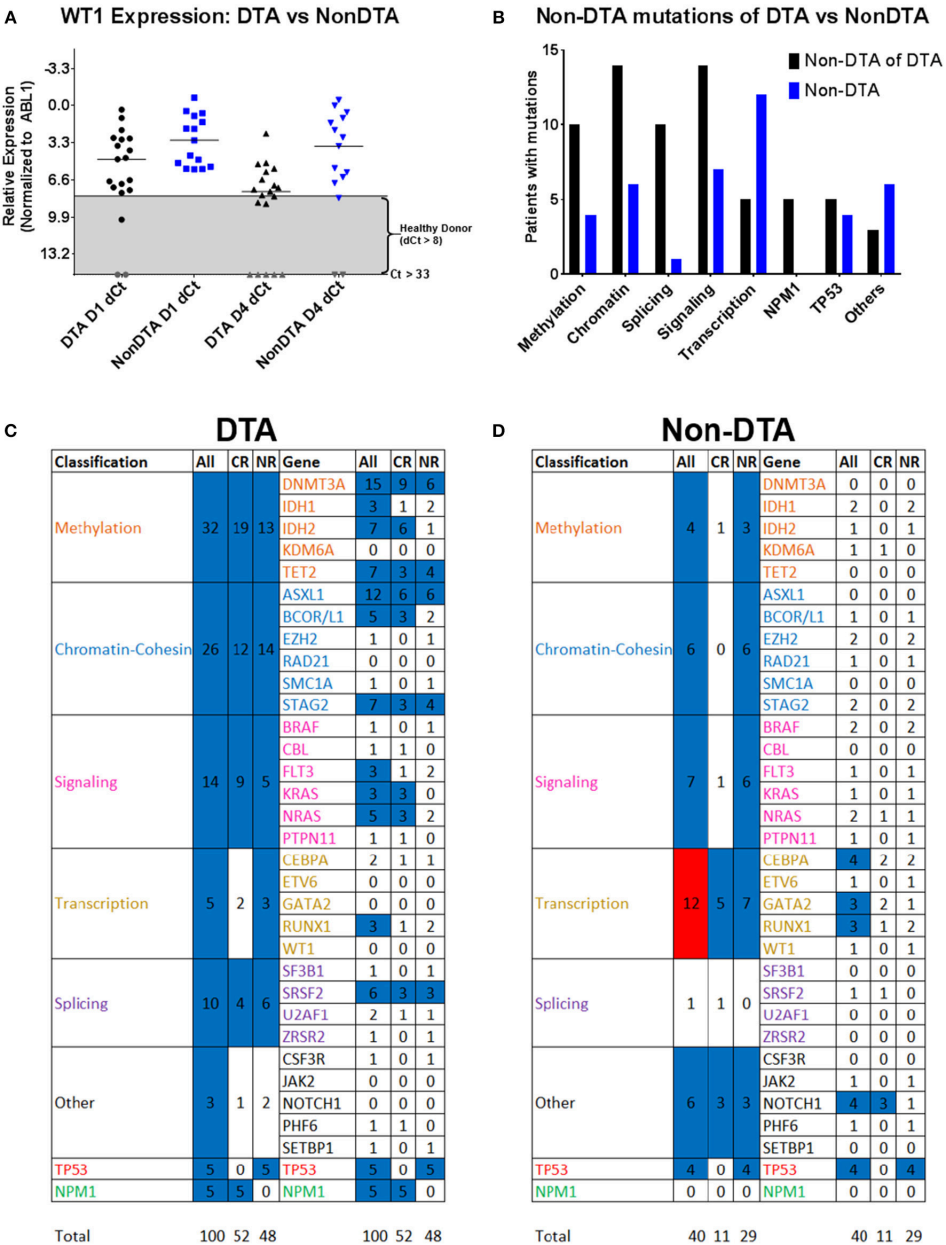
WT1 expression and gene variants in DTA and non-DTA patients. Patients were separated into groups based on detection of at least one DTA (DNMT3A, TET2, and ASXL1) mutation. The 34 genes in which variants were identified were classified into eight functional groups and Day 1 vs. 4 VAFs were analyzed with the Wilcoxon signed rank test. 162 coding variants were condensed into 140 by averaging the VAFs of multiple variants in the same gene in the same patient. The DTA group had 100 variants and the non-DTA group had 40 variants. **(A)** WT1 expression was higher in non-DTA patients compared to DTA patients. Out of the 34 patients with complete WT1 expression data, 19 patients were DTA patients and 15 were non-DTA. Here, non-DTA patients had higher relative WT1 expression than DTA patients at both D1 (median relative expression 3.07 compared to 4.75, *P* < 0.05) and D4 (median relative expression 3.59 compared to 7.60, *P* < 0.05). **(B)** Variants observed in DTA vs. non-DTA patients. NPM1 mutations were detected in DTA patients only and more DTA patients have variants in all functional gene groups compared to non-DTA patients except for transcription genes. **(C,D)** Heatmap of statistical significance between D1 vs. D4 VAFs of mutated genes in functional groups for both DTA and non-DTA patients. The “All Transcription” group in non-DTA patients was significant (*P* < 0.05, red) and NPM1, detected only in CR DTA patients, was marginally significant (blue, *P* = 0.0625). The remaining functional groups and individual genes were either nonsignificant (blue) or had too few data points to be analyzed (white). DTA, mutations in DNMT3A, TET2, or ASXL1; qPCR, quantitative real-time PCR; NGS: Targeted DNA sequencing. Gray, undetectable; Gray box indicates healthy donor range; VAF, variant allele frequency; CR, complete remission; NR, non-responder; DX, Day X.

Somatic mutations in *DNMT3A, TET2*, and *ASXL1* (referred to as “DTA” mutations) are commonly found in AML patients but are also seen in clinically asymptomatic individuals with increased prevalence with aging (27–30). Mutations in these genes are known to not be useful in measuring residual disease in AML (6, 12). Patients with and without DTA mutations were therefore analyzed separately (Figure 7). Overall, 27 patients (60%) had a DTA mutation, and this observation was consistent between the NHLBI relapsed/refractory (median age: 52) and Duke newly diagnosed (median age: 63) AML cohorts. DTA patients expressed significantly lower levels of *WT1* than non-DTA patients (median dCT of 4.8 compared to 3.1 *P* < 0.05) but had greater decrease in *WT1* levels by day 4 (median dCT 7.6 vs. 3.6, *P* < 0.05). 48% of DTA patients achieved CR compared with 33% of non-DTA patients. *NPM1* mutations (*n* = 5) were seen exclusively in DTA patients while *TP53* mutations were seen in both DTA (*n* = 5) and non-DTA (*n* = 4) patients. Only the transcription-related gene class, in non-DTA patients, showed significant difference between day 1 and 4 VAF levels (Figure 7).

### Tracking Post-treatment MRD With Targeted DNA Sequencing During Remission

**Figure 8:**
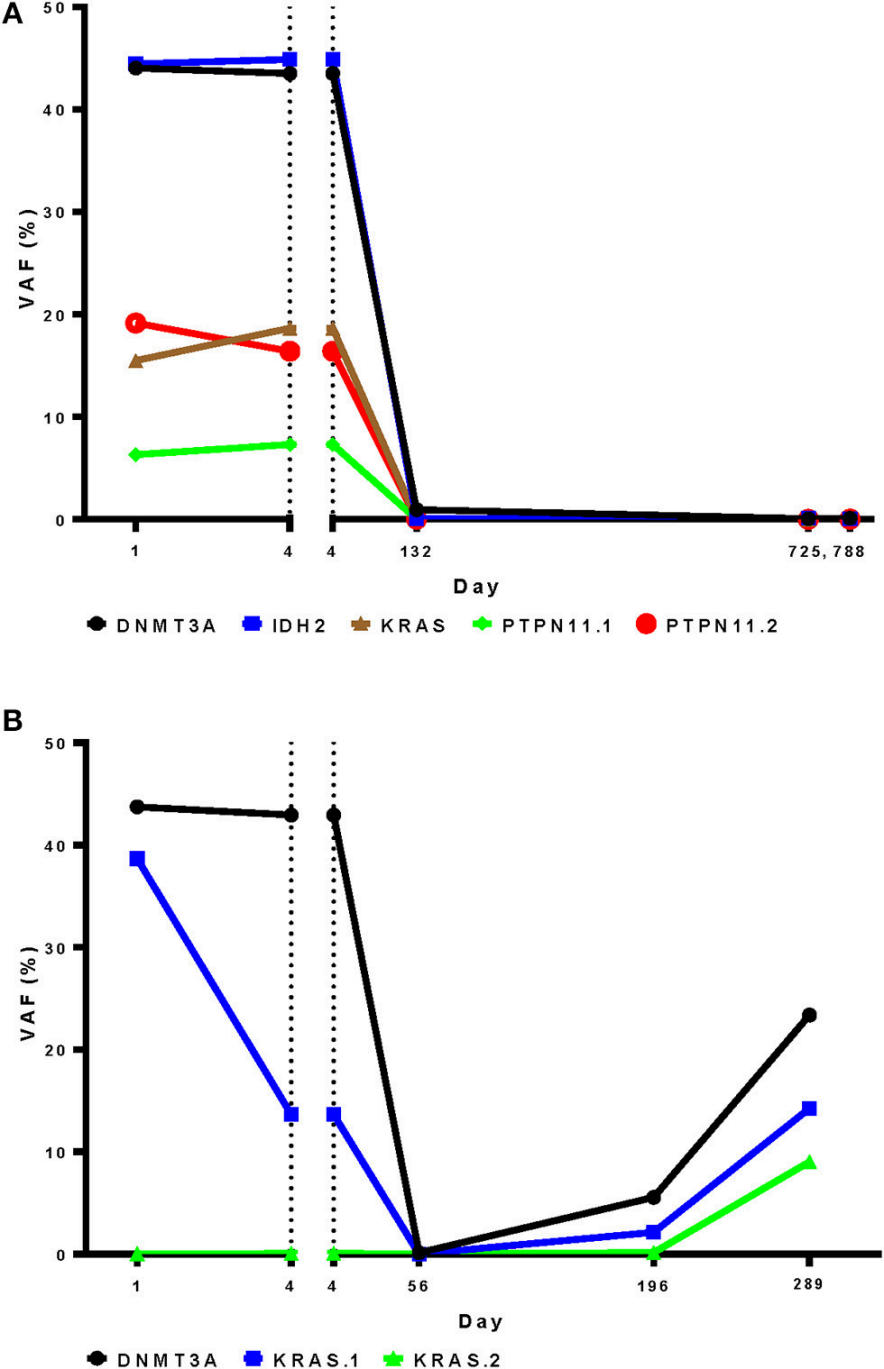
Targeted DNA sequencing for residual disease may be more informative after, rather than during, initial chemotherapy. A DNA sequencing panel customized to patient-specific variants was used to for analysis of longitudinal blood samples from during and after induction therapy in two patients who achieved complete remission. **(A)** Variant frequency was unchanged in the first patient between day 1 and 4 during therapy, despite achievement of a durable complete remission **(B)**. In the second patient a *DNMT3A* variant changed little during therapy, in contrast to a *KRAS* variant that was markedly reduced. Relapse occurred on day 154. Both mutations were detected In blood from day 196 followed by the emergence of an second *KRAS* mutation on day 289. VAF, variant allele frequency.

Given the inability of targeted DNA sequencing of blood early during therapy to predict response to a cycle of intensive chemotherapy, we also investigated the utility of this technique in predicting post-remission relapse. Longitudinal blood samples were available from two patients from this cohort both of whom achieved CR. The first patient had no change in the ratios of wild type to mutated sequence of five genes between day 1 and 4 of therapy, despite decreasing WBC count from 60,000 to 10,000/μl during this period and subsequently achievement of a durable CR. Mutation levels remained negligible however during a durable remission lasting at least two years (Figure 8A). In the second patient, detectable *KRAS* mutant in blood decreased during the first 4 days of therapy while the *DNMT3A* mutant remained stable. During remission however both mutations were undetectable, returning at the time of relapse together with the emergence of a second *KRAS* mutation (Figure 8B).

### DNA Sequencing From Blood vs. Bone Marrow Samples

**Figure 9:**
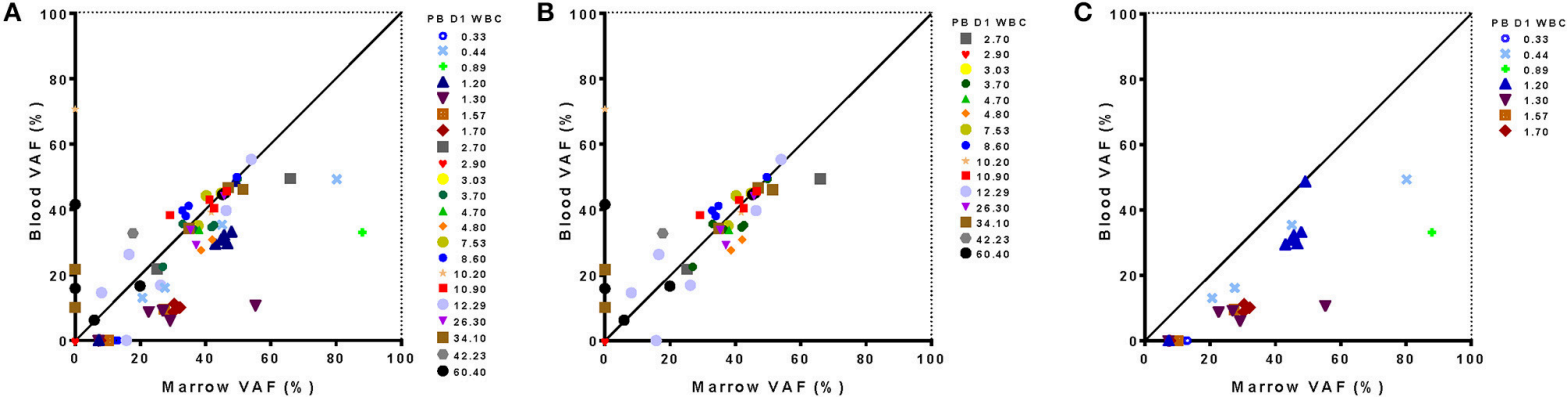
Relationship between sequence variants detected from blood and marrow samples in AML patients. Results of targeted DNA sequencing at day 1 of treatment show good concordance with outliers associated to extremes of white blood cell (WBC) count in blood. Variant allele frequency detected from in blood and marrow is shown for all 22 patients evaluated **(A)**, just those patients with WBC >2,500/μl **(B)** or just those patients with WBC <2,500/μl in blood **(C)**. Leukopenic patients had lower VAF in blood for variants identified from marrow. One patient had no detectable mutations in either blood or marrow. PB D1 WBC, White blood cell count in blood on Day 1; VAF, variant allele frequency.

In a subset of 22 patients confirmatory sequencing was also performed on pre-treatment bone marrow samples (10/10 NHLBI, 12/35 Duke). Concordance between the number of variants identified and the VAF of each detected variant in blood compared with bone marrow was assessed (Figure 9A). In the 15 patients with a pre-treatment WBC of at least 2,500/μl only a single variant identified from bone marrow was not detected from blood (of 41 variants identified in total, i. e., 98%). Notably, there was good correlation in the VAF determined for each variant from both tissue sources in these patients. Conversely, five variants were identified only from blood and not from bone marrow.

Interestingly, the presence or absence of mutations in the blood vs. bone marrow was often correlated with WBC. Two patients with high pre-treatment WBC (34,100 and 60,400/μl) accounted for four of the five variants variants observed in the blood but not the bone marrow (Figure 9B). Likewise, for 7 patients with pre-treatment WBC <2,500/μl, only 20 out of a total of 27 variants detected in bone marrow were also detected from blood (74%), with the consequence that 5 of 7 leukopenic AML patients had an incomplete mutational characterization by DNA sequencing when using blood alone (Figure 9C). Those variants found in marrow but not identified in blood could however be identified in the raw sequencing data but were filtered out by the 5% VAF threshold for variant calling. For all 20 variants identified in both tissues the VAF was lower in blood than marrow in these leukopenic patients.

## Discussion

As we strive to personalize treatments to cancer patients, both in terms of the genetic basis of their cancer and their response to therapy, there is a great interest in earlier assessments of disease burden and characteristics (31, 32). Acute myeloid leukemia (AML) offers a unique opportunity to study the validity of blood-based assessments of residual tumor, as both the primary site of disease (bone marrow) and blood are repeatedly sampled as part of the clinical standard of care. It is increasingly recognized that blood, except in cases of leukopenia or low circulating blast count, may substitute for marrow examination in some circumstances for morphology, cytogenetics, and molecular testing in AML patients (33–35). We show here that blood, even after 3 days of intensive cytotoxic chemotherapy, can be used to identify most of the MDS/AML-associated DNA variants detectable by targeted sequencing in pre-treatment bone marrow aspirate, providing that pre-treatment white blood cell count was within or above normal range.

Complete remission is a necessary, but often insufficient, step toward long-term cure in AML. Given that the outcome of patients with relapsed and refractory AML is generally poor (36, 37) there is great interest in early interim assessments of likely response to optimize therapy in AML. While the role of bone marrow examination on day 14 of induction therapy remains unclear (35, 38–41), there has been considerable interest in kinetics of early blast clearance in blood during induction therapy as a prognostic factor (40, 42–44). Persistence of variants detected by targeted sequencing in AML patients in CR after treatment has significant independent prognostic value for both relapse and survival (6). It was therefore intriguing to consider if such molecular assessments, performed during cytotoxic therapy and prior to any response assessment, could offer a “real-time” evaluation of treatment efficacy and extremely early identification of treatment failure. We show that molecular testing of blood on treatment day 4, by either qPCR assessment of *WT1* expression or DNA sequencing for common MDS/AML variants, is not predictive of clinical response to intensive cytotoxic therapy in AML. Surprisingly, all variants found by DNA sequencing in blood on day 1 remained detectable on the fourth day of intensive chemotherapy. Remarkably the ratio of mutated to wild-type sequence was often maintained during this therapy despite considerable reductions in white blood cell count. This finding of stability was not limited to potential germline mutants or to variants in *DNMT3A, TET2*, and *ASXL1*. The observed stability in VAF during cytotoxic therapy may suggest a limited therapeutic index for clones circulating in blood containing these mutations, although similar studies in patients receiving highly effective and specific therapy would be needed to prove this definitively. The kinetics of *NPM1* and *TP53* variant burden early during chemotherapy however did appear to exhibit consistent trends during therapy in this cohort and are markers potentially worthy of future study. Ultimately DNA sequencing may have greater utility for tracking of AML MRD post-treatment rather than predicting response during therapy.

This study has several limitations. Firstly, we did not perform germline sequencing to allow categorization of identified variants by DNA sequencing as somatic. This was intentional, given the translational nature of this study, we wished to replicate testing as commonly performed in clinical practice. Importantly germline mutations would not be expected to change during chemotherapy so they would not be informative for, or contribute to, variant kinetic analysis. Additionally, the majority of variants identified had a VAF of <40%, and 42 of 43 assessed patients had at least one variant with VAF <40% detectable in blood at baseline. Secondly, AML represents a wide range of myeloid malignancies with many patterns of genetic etiology whereas we used a targeted sequencing panel designed to detect just 49 commonly mutated genes or gene regions in MDS/AML (Table S3). While more comprehensive approaches have been used to characterize potential genetic variants associated with the leukemic clone that may be detectable before and after therapy (11), we felt assessment of the most recurrently observed mutated regions was most easily translatable. Finally, we demonstrate consistent observations in two independent cohorts, at two different stages of disease treated with different intensive chemotherapy regimens. Given the genetic heterogeneity of this disease however it is possible that larger cohorts of patients would identify additional trends and will certainly be needed to quantify any benefit associated with tracking the two targets, *TP53* and *NPM1*, we identify as potential candidates for monitoring during intensive therapy.

In conclusion, our study demonstrates that molecular testing of peripheral blood during the first 3 days of AML intensive chemotherapy does not appear to be predictive of clinical response. Indeed, we show that the majority of variants identified prior to treatment are still present and often at similar ratios of mutant to wild-type often despite considerable cytotoxic effect of therapy. Validation in a larger cohort is needed to confirm the utility of monitoring *NPM1* and *TP53* variant kinetics in blood early during AML treatment in addition to their current use as pre-treatment predictive markers. Consistent with reports using other modalities we show that blood may substitute for bone marrow for targeted DNA sequencing in AML patients, although this approach may be suboptimal in those with leukopenia pre-treatment. Longitudinal assessment of molecular MRD during follow-up time points after completion of initial cytotoxic induction therapy may have greater clinical utility than evaluation of very early time points during AML treatment.

## Ethics Statement

Ten subjects were recruited to clinical protocol (NCT02527447). All subjects gave written informed consent in accordance with the Declaration of Helsinki. The protocol was approved by the NHLBI IRB. Samples from thirty-five patients were collected on a Duke IRB approved protocol and transferred to NIH as coded clinically annotated samples via a material transfer agreement judged exempt from NIH IRB by the NIH OHSRP.

## Author Contributions

HW, AS, and CH, contributed conception and design of the study. HW, KL, SS, GR, MG, MM, JTa, and LD performed laboratory research. AS, SS, DR, NR, JTh, CD, KR, CL, and CH performed clinical research. HW and D-YK performed the statistical analysis. HW wrote the first draft of the manuscript. All authors contributed to manuscript revision, read, and approved the submitted version.

### Conflict of Interest Statement

CH receives laboratory research funding from Merck and Sellas. The remaining authors declare that the research was conducted in the absence of any commercial or financial relationships that could be construed as a potential conflict of interest.

## References

[1] Dohner H Estey E Grimwade D Amadori S Appelbaum FR Buchner T. Diagnosis and management of AML in adults: 2017 ELN recommendations from an international expert panel. Blood (2017) 129:424–47. 10.1182/blood-2016-08-733196 27895058 PMC5291965

[2] Schuurhuis GJ Heuser M Freeman S Bene MC Buccisano F Cloos J. Minimal/measurable residual disease in AML: a consensus document from the European LeukemiaNet MRD Working Party. Blood (2018) 131:1275–91. 10.1182/blood-2017-09-801498 29330221 PMC5865231

[3] Arber DA Borowitz MJ Cessna M Etzell J Foucar K Hasserjian RP. Initial diagnostic workup of acute leukemia: guideline from the College of American Pathologists and the American Society of Hematology. Arch Pathol Lab Med. (2017):1342–93. 10.5858/arpa.2016-0504-CP 28225303

[4] Hourigan CS Karp JE. Minimal residual disease in acute myeloid leukaemia. Nat Rev Clin Oncol. (2013) 10:460–71. 10.1038/nrclinonc.2013.100 23799371 PMC4163748

[5] Zhou Y Othus M Walter RB Estey EH Wu D Wood BL. Deep NPM1 sequencing following allogeneic hematopoietic cell transplantation improves risk assessment in adults with NPM1-mutated AML. Biol Blood Marrow Transplant. (2018) 24:1615–20. 10.1016/j.bbmt.2018.04.017 29684564

[6] Jongen-Lavrencic M Grob T Hanekamp D Kavelaars FG Al Hinai A Zeilemaker A. Molecular minimal residual disease in acute myeloid leukemia. N Engl J Med. (2018) 378:1189–99. 10.1056/NEJMoa1716863 29601269

[7] Malmberg EB Stahlman S Rehammar A Samuelsson T Alm SJ Kristiansson E. Patient-tailored analysis of minimal residual disease in acute myeloid leukemia using next-generation sequencing. Eur J Haematol. (2017) 98:26–37. 10.1111/ejh.12780 27197529

[8] Gaksch L Kashofer K Heitzer E Quehenberger F Daga S Hofer S. Residual disease detection using targeted parallel sequencing predicts relapse in cytogenetically normal acute myeloid leukemia. Am J Hematol. (2018) 93:23–30. 10.1002/ajh.24922 28960408

[9] Morita K Kantarjian HM Wang F Yan Y Bueso-Ramos C Sasaki K. Clearance of somatic mutations at remission and the risk of relapse in acute myeloid leukemia. J Clin Oncol. (2018) 36:1788–97. 10.1200/JCO.2017.77.6757 29702001 PMC6008108

[10] Rothenberg-Thurley M Amler S Goerlich D Kohnke T Konstandin NP Schneider S. Persistence of pre-leukemic clones during first remission and risk of relapse in acute myeloid leukemia. Leukemia (2018) 32:1598–608. 10.1038/s41375-018-0034-z 29472724 PMC6035153

[11] Klco JM Miller CA Griffith M Petti A Spencer DH Ketkar-Kulkarni S. Association between mutation clearance after induction therapy and outcomes in acute myeloid leukemia. JAMA (2015) 314:811–22. 10.1001/jama.2015.9643 26305651 PMC4621257

[12] Debarri H Lebon D Roumier C Cheok M Marceau-Renaut A Nibourel O. IDH1/2 but not DNMT3A mutations are suitable targets for minimal residual disease monitoring in acute myeloid leukemia patients: a study by the Acute Leukemia French Association. Oncotarget (2015) 6:42345–53. 10.18632/oncotarget.5645 26486081 PMC4747230

[13] Dillon LW Hayati S Roloff GW Tunc I Pirooznia M Mitrofanova A. Targeted RNA-sequencing for the quantification of measurable residual disease in acute myeloid leukemia. Haematologica (2018). [Epub ahead of print]. 10.3324/haematol.2018.203133 30171026 PMC6355494

[14] Thol F Gabdoulline R Liebich A Klement P Schiller J Kandziora C. Measurable residual disease monitoring by NGS before allogeneic hematopoietic cell transplantation in AML. Blood (2018) 132:1703–13. 10.1182/blood-2018-02-829911 30190321 PMC7116653

[15] Hourigan CS Gale RP Gormley NJ Ossenkoppele GJ Walter RB. Measurable residual disease testing in acute myeloid leukaemia. Leukemia (2017) 31:1482–90. 10.1038/leu.2017.113 28386105

[16] Terwijn M van Putten WL Kelder A van der Velden VH Brooimans RA Pabst T. High prognostic impact of flow cytometric minimal residual disease detection in acute myeloid leukemia: data from the HOVON/SAKK AML 42A study. J Clin Oncol. (2013) 31:3889–97. 10.1200/JCO.2012.45.9628 24062400

[17] Ivey A Hills RK Simpson MA Jovanovic JV Gilkes A Grech A. Assessment of minimal residual disease in standard-risk AML. N Engl J Med. (2016) 374:422–33. 10.1056/NEJMoa1507471 26789727

[18] Freeman SD Hills RK Virgo P Khan N Couzens S Dillon R. Measurable residual disease at induction redefines partial response in acute myeloid leukemia and stratifies outcomes in patients at standard risk without NPM1 mutations. J Clin Oncol. (2018) 36:1486–97. 10.1200/JCO.2017.76.3425 29601212 PMC5959196

[19] Buckley SA Wood BL Othus M Hourigan CS Ustun C Linden MA. Minimal residual disease prior to allogeneic hematopoietic cell transplantation in acute myeloid leukemia: a meta-analysis. Haematologica (2017) 102:865–73. 10.3324/haematol.2016.159343 28126965 PMC5477605

[20] Araki D Wood BL Othus M Radich JP Halpern AB Zhou Y. Allogeneic hematopoietic cell transplantation for acute myeloid leukemia: time to move toward a minimal residual disease-based definition of complete remission? J Clin Oncol. (2016) 34:329–36. 10.1200/JCO.2015.63.3826 26668349 PMC4872033

[21] Hourigan CS Goswami M Battiwalla M Barrett AJ Sheela S Karp JE. When the minimal becomes measurable. J Clin Oncol. (2016) 34:2557–8. 10.1200/JCO.2016.67.6395 27185839

[22] Cilloni D Renneville A Hermitte F Hills RK Daly S Jovanovic JV. Real-time quantitative polymerase chain reaction detection of minimal residual disease by standardized WT1 assay to enhance risk stratification in acute myeloid leukemia: a European LeukemiaNet study. J Clin Oncol. (2009) 27:5195–201. 10.1200/JCO.2009.22.4865 19752335

[23] Goswami M Hensel N Smith BD Prince GT Qin L Levitsky HI. Expression of putative targets of immunotherapy in acute myeloid leukemia and healthy tissues. Leukemia (2014) 28:1167–70. 10.1038/leu.2014.14 24472813 PMC4013200

[24] Goswami M McGowan KS Lu K Jain N Candia J Hensel NF. A multigene array for measurable residual disease detection in AML patients undergoing SCT. Bone Marrow Transplantat. (2015) 50:642–51. 10.1038/bmt.2014.326 25665046 PMC4424111

[25] Cancer Genome Atlas Research Network Ley TJ Miller C Ding L Raphael BJ Mungall AJ. Genomic and epigenomic landscapes of adult *de novo* acute myeloid leukemia. N Engl J Med. (2013) 368:2059–74. 10.1056/NEJMoa1301689 23634996 PMC3767041

[26] Papaemmanuil E Gerstung M Bullinger L Gaidzik VI Paschka P Roberts ND. Genomic classification and prognosis in acute myeloid leukemia. N Engl J Med. (2016) 374:2209–21. 10.1056/NEJMoa1516192 27276561 PMC4979995

[27] Genovese G Kahler AK Handsaker RE Lindberg J Rose SA Bakhoum SF. Clonal hematopoiesis and blood-cancer risk inferred from blood DNA sequence. N Engl J Med. (2014) 371:2477–87. 10.1056/NEJMoa1409405 25426838 PMC4290021

[28] Zink F Stacey SN Norddahl GL Frigge ML Magnusson OT Jonsdottir I. Clonal hematopoiesis, with and without candidate driver mutations, is common in the elderly. Blood (2017) 130:742–52. 10.1182/blood-2017-02-769869 28483762 PMC5553576

[29] Shlush LI. Age-related clonal hematopoiesis. Blood (2018) 131:496–504. 10.1182/blood-2017-07-746453 29141946

[30] Jaiswal S Fontanillas P Flannick J Manning A Grauman PV Mar BG. Age-related clonal hematopoiesis associated with adverse outcomes. N Engl J Med. (2014) 371:2488–98. 10.1056/NEJMoa1408617 25426837 PMC4306669

[31] Ravandi F Walter RB Freeman SD. Evaluating measurable residual disease in acute myeloid leukemia. Blood Adv. (2018) 2:1356–66. 10.1182/bloodadvances.2018016378 29895626 PMC5998930

[32] Grimwade D Freeman SD. Defining minimal residual disease in acute myeloid leukemia: which platforms are ready for “Prime Time”? Blood (2014) 124:3345–55. 10.1182/blood-2014-05-577593. 25049280

[33] Weinkauff R Estey EH Starostik P Hayes K Huh YO Hirsch-Ginsberg C. Use of peripheral blood blasts vs bone marrow blasts for diagnosis of acute leukemia. Am J Clin Pathol. (1999) 111:733–40. 10.1093/ajcp/111.6.733 10361507

[34] Tong WG Sandhu VK Wood BL Hendrie PC Becker PS Pagel JM. Correlation between peripheral blood and bone marrow regarding FLT3-ITD and NPM1 mutational status in patients with acute myeloid leukemia. Haematologica (2015) 100:e97–8. 10.3324/haematol.2014.118422 25527567 PMC4349287

[35] Percival ME Lai C Estey E Hourigan CS. Bone marrow evaluation for diagnosis and monitoring of acute myeloid leukemia. Blood Rev. (2017) 31:185–92. 10.1016/j.blre.2017.01.003 28190619 PMC5513766

[36] Breems DA Van Putten WL Huijgens PC Ossenkoppele GJ Verhoef GE Verdonck LF. Prognostic index for adult patients with acute myeloid leukemia in first relapse. J Clin Oncol. (2005) 23:1969–78. 10.1200/JCO.2005.06.027 15632409

[37] Ramos NR Mo CC Karp JE Hourigan CS. Current approaches in the treatment of relapsed and refractory acute myeloid leukemia. J Clin Med. (2015) 4:665–95. 10.3390/jcm4040665 25932335 PMC4412468

[38] Morris TA DeCastro CM Diehl LF Gockerman JP Lagoo AS Li Z. Re-induction therapy decisions based on day 14 bone marrow biopsy in acute myeloid leukemia. Leukemia Res. (2013) 37:28–31. 10.1016/j.leukres.2012.09.016 23046833 PMC3753071

[39] Hussein K Jahagirdar B Gupta P Burns L Larsen K Weisdorf D. Day 14 bone marrow biopsy in predicting complete remission and survival in acute myeloid leukemia. Am J Hematol. (2008) 83:446–50. 10.1002/ajh.21133 18247382

[40] Yanada M Borthakur G Ravandi F Bueso-Ramos C Kantarjian H Estey E. Kinetics of bone marrow blasts during induction and achievement of complete remission in acute myeloid leukemia. Haematologica (2008) 93:1263–5. 10.3324/haematol.12825 18519513

[41] Yezefski T Xie H Walter R Pagel J Becker PS Hendrie P. Value of routine ‘day 14’ marrow exam in newly diagnosed AML. Leukemia (2015) 29:247–9. 10.1038/leu.2014.268 25204570

[42] Kern W Haferlach T Schoch C Loffler H Gassmann W Heinecke A. Early blast clearance by remission induction therapy is a major independent prognostic factor for both achievement of complete remission and long-term outcome in acute myeloid leukemia: data from the German AML Cooperative Group (AMLCG) 1992 Trial. Blood (2003) 101:64–70. 10.1182/blood-2002-02-0532 12393605

[43] Gianfaldoni G Mannelli F Baccini M Antonioli E Leoni F Bosi A. Clearance of leukaemic blasts from peripheral blood during standard induction treatment predicts the bone marrow response in acute myeloid leukaemia: a pilot study. Br J Haematol. (2006) 134:54–7. 10.1111/j.1365-2141.2006.06100.x 16803567

[44] Vainstein V Buckley SA Shukron O Estey EH Abkowitz JL Wood BL. Rapid rate of peripheral blood blast clearance accurately predicts complete remission in acute myeloid leukemia. Leukemia (2014) 28:713–6. 10.1038/leu.2013.341 24240201

