# Supplementary material for: Molecular Measurable Residual Disease Testing of Blood During AML Cytotoxic Therapy for Early Prediction of Clinical Response
Source: Front Oncol. 2019 Jan 15;8:669. doi: 10.3389/fonc.2018.00669 (PMC6341003; doi:10.3389/fonc.2018.00669)
Supplement: Supplementary file 1 [file Data_Sheet_1.PDF]

*Supplementary Materials for:*

**Molecular measurable residual disease testing of blood during AML cytotoxic therapy for early prediction of clinical response.**

[1]Hong Yuen Wong, [2]Anthony D. Sung, [1]Katherine E. Lindblad, [1]Sheenu Sheela, [1]Gregory W. Roloff, [2]David Rizzieri, [1]Meghali Goswami, [1]Matthew P. Mulé, [1]Nestor R. Ramos, [1]Jingrong Tang, [1]Julie Thompson, [1]Christin B. Destefano,[2]Kristi Romero, [1]Laura W. Dillon, [3]Dong-Yun Kim, [1]Catherine Lai, [1]Christopher S. Hourigan

[1]Laboratory of Myeloid Malignancies Section, Hematology Branch, National Heart, Lung and Blood Institute, Bethesda, MD;

[2]Duke University School of Medicine, Durham, NC;

[3]Office of Biostatistics Research, Division of Cardiovascular Sciences, National Heart, Lung and Blood Institute, Bethesda, MD

**Contents:**

Figure S1

Tables S1 - S3

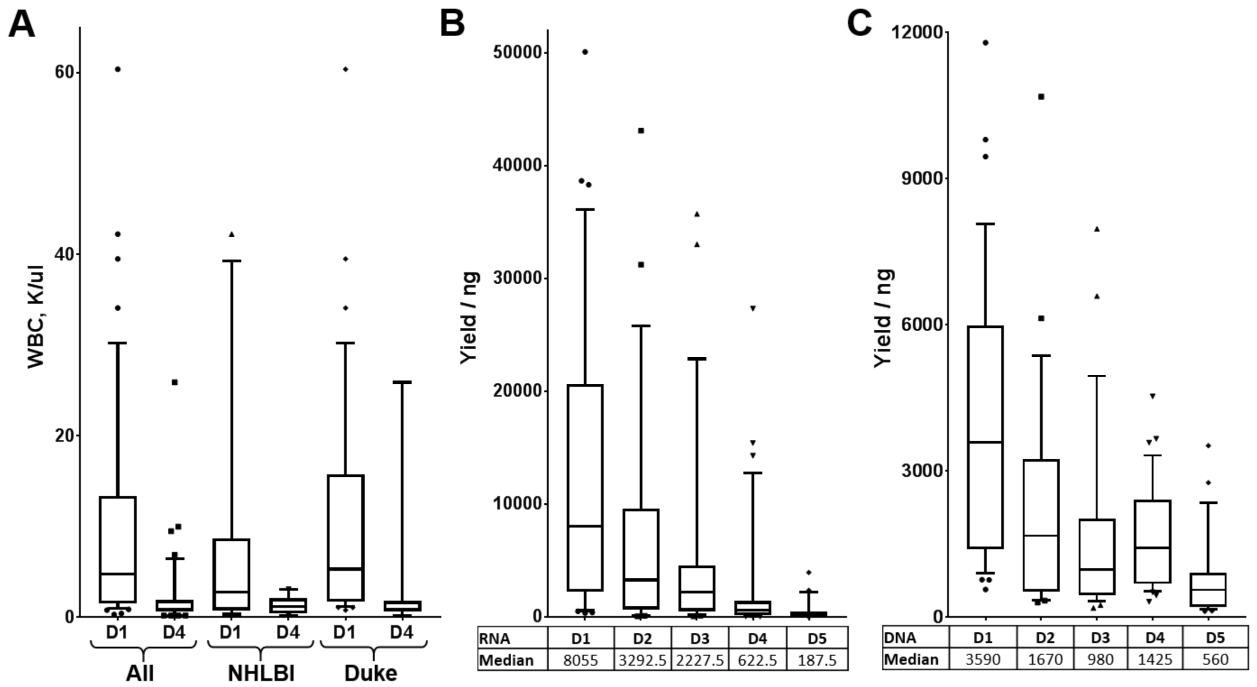


| RNA | D1 | D2 | D3 | D4 | D5 |
|---|---|---|---|---|---|
| Median | 8055 | 3292.5 | 2227.5 | 622.5 | 187.5 |

| DNA | D1 | D2 | D3 | D4 | D5 |
|---|---|---|---|---|---|
| Median | 3590 | 1670 | 980 | 1425 | 560 |

**D**

| Duke Speedvac Record | n = * | D1 Yield Median (Range) / ng | D1 Speedvac Success | D4 Yield Median (Range) / ng | D4 Speedvac Success | Samples Analyzed |
|---|---|---|---|---|---|---|
| All Samples | 35 | 8055 (367.5 - 50070) | 9 out of 9 | 660 (22.5 - 27322.5) | 17 out of 28 | 24 |
| Sufficient RNA | 7 | 30232.5 (8370 - 38655) | NA | 9180 (1830 - 27322.5) | NA | 7 |
| Insufficient RNA at D4 | 19 | 11730 (2257.5 - 50070) | NA | 517.5 (22.5 - 1440) | 13 out of 19 | 13 |
| Insufficient RNA at D1 and D4 | 9 | 705 (367.5 - 2357.5) | 9 out of 9 | 180 (52.5 - 1387.5) | 4 out of 9 | 4 |

*n = number of patients, each patient has paired D1 and D4 RNA samples.

**Figure S1. Feasibility of molecular testing during AML cytotoxic therapy.**

**A)** Peripheral blood white blood cell **(**WBC) counts (K/ul) pre-treatment. The median WBC on Day 1 in All, NHLBI, and Duke cohorts were 4.8, 2.8, and 5.3, respectively; the median WBC counts on Day 4 were 0.9, 1.17, and 0.9, respectively.

**B&C)** Day 1 to 5 nucleic acid yields from Duke patients (B: RNA, C: DNA) . Yields dropped during treatment so that there was insufficient RNA beyond Day 4 for molecular testing. Boxplots show median lines with a range of $10^{th}$ and $90^{th}$ percentiles.

**D)** Concentration of Duke RNA samples. 37 of 70 extracted RNA samples (9 from day 1, 26 from day 4) were inadequately concentrated at the time of isolation. Subsequent concentration was successful for 26 of the 37 samples, allowing 24 of the 34 patients to have both day 1 and 4 RNA to be tested for *WT1* and *ABL1* expression by qPCR.

**Table S1. Demographics of 45 patients**

| NHLBI | Age Range | Median Age | Mean Age | Male | Female | ELN Risk Stratification* Favorable | Intermediate | Adverse | Unclassified | Total |
|---|---|---|---|---|---|---|---|---|---|---|
| CR | 39 to 66 | 58 | 55 | 1 | 3 | 1 | 2 | 1 | 0 | 4 |
| NR | 23 to 64 | 52 | 50 | 4 | 2 | 0 | 1 | 5 | 0 | 6 |
| All | 23 to 66 | 52 | 52 | 5 | 5 | 1 | 3 | 6 | 0 | 10 |

| Duke | Age Range | Median Age | Mean Age | Male | Female | ELN Risk Stratification Favorable | Intermediate | Adverse | Unclassified | Total |
|---|---|---|---|---|---|---|---|---|---|---|
| CR | 43 to 77 | 63 | 60 | 7 | 8 | 10 | 3 | 1 | 1 | 15 |
| NR | 30 to 78 | 64 | 59 | 10 | 10 | 1 | 7 | 8 | 4 | 20 |
| All | 30 to 78 | 63 | 60 | 17 | 18 | 11 | 10 | 9 | 5 | 35 |

| Total | Age Range | Median Age | Mean Age | Male | Female | ELN Risk Stratification Favorable | Intermediate | Adverse | Unclassified | Total |
|---|---|---|---|---|---|---|---|---|---|---|
| CR | 39 to 77 | 63 | 59 | 8 | 11 | 11 | 5 | 2 | 1 | 19 |
| NR | 23 to 78 | 62 | 57 | 14 | 12 | 1 | 8 | 13 | 4 | 26 |
| All | 23 to 78 | 62 | 58 | 22 | 23 | 12 | 13 | 15 | 5 | 45 |

*Risk stratification on the basis of cytogenetics and molecular features in patients with refractory or relapsed AML may not be clinically informative and is shown here only for reader's interest.

**Table S2. Clinical characteristics of all 45 patients**

| Unique ID | Age | Gender | Risk Stratification | Status | Treatment | Response |
|---|---|---|---|---|---|---|
| **NHLBI (4 CR; 6 NR)** | | | | | | |
| 01 | 50 | F | Intermediate | Relapsed | EMA | CR |
| 02 | 65 | F | Adverse | Refractory | EMA | CR |
| 03 | 39 | F | Favorable | Relapsed | EMA | CR |
| 04 | 66 | M | Intermediate | Relapsed | EMA | CR |
| 05 | 45 | F | Adverse | Refractory | EMA | NR |
| 06 | 53 | M | Adverse | Refractory | G-CLAC | NR |
| 07 | 50 | M | Adverse | Refractory | EMA | NR |
| 08 | 23 | M | Intermediate | Refractory | EMA | NR |
| 09 | 64 | M | Adverse | Refractory | EMA | NR |
| 10 | 62 | F | Adverse | Refractory | EMA | NR |
| **Duke (15 CR; 20 NR)** | | | | | | |
| 11 | 47 | M | Favorable | *De novo* | 7 + 3 | CR |
| 12 | 65 | M | Favorable | *De novo* | 7 + 3 | CR |
| 13 | 69 | M | Intermediate | *De novo* | 7 + 3 | CR |
| 14 | 77 | M | Intermediate | *De novo* | 7 + 3 | CR |
| 15 | 68 | M | Favorable | *De novo* | 7 + 3 | CR |
| 16 | 69 | F | Favorable | *De novo* | 7 + 3 | CR |
| 17 | 58 | F | Favorable | *De novo* | 7 + 3 | CR |
| 18 | 68 | F | Favorable | *De novo* | 7 + 3 | CR |
| 19 | 59 | M | Favorable | *De novo* | 7 + 3 | CR |
| 20 | 50 | F | Favorable | *De novo* | 7 + 3 | CR |
| 21 | 43 | F | Adverse | *De novo* | 7 + 3 | CR |
| 22 | 63 | M | Unclassified | *De novo* | 7 + 3 | CR |
| 23 | 56 | F | Intermediate | *De novo* | 7 + 3 | CR |
| 24 | 68 | F | Favorable | *De novo* | 7 + 3 | CR |
| 25 | 46 | F | Favorable | *De novo* | 7 + 3 | CR |
| 26 | 59 | F | Intermediate | *De novo* | 7 + 3 | NR |
| 27 | 63 | F | Adverse | *De novo* | 7 + 3 | NR |
| 28 | 70 | M | Adverse | *De novo* | 7 + 3 | NR |
| 29 | 68 | F | Adverse | *De novo* | 7 + 3 | NR |
| 30 | 65 | M | Intermediate | *De novo* | 7 + 3 | NR |
| 31 | 66 | F | Intermediate | *De novo* | 7 + 3 | NR |
| 32 | 42 | M | Adverse | *De novo* | 7 + 3 | NR |
| 33 | 30 | F | Adverse | *De novo* | 7 + 3 | NR |
| 34 | 64 | F | Favorable | *De novo* | 7 + 3 | NR |
| 35 | 78 | F | Adverse | *De novo* | 7 + 3 | NR |
| 36 | 34 | M | Unclassified | *De novo* | 7 + 3 | NR |
| 37 | 43 | F | Intermediate | *De novo* | 7 + 3 | NR |
| 38 | 55 | M | Intermediate | *De novo* | 7 + 3 | NR |
| 39 | 72 | M | Intermediate | *De novo* | 7 + 3 | NR |
| 40 | 76 | M | Unclassified | *De novo* | 7 + 3 | NR |
| 41 | 59 | F | Intermediate | *De novo* | 7 + 3 | NR |
| 42 | 72 | M | Unclassified | *De novo* | 7 + 3 | NR |
| 43 | 50 | M | Unclassified | *De novo* | 7 + 3 | NR |
| 44 | 62 | M | Adverse | *De novo* | 7 + 3 | NR |
| 45 | 72 | M | Adverse | *De novo* | 7 + 3 | NR |

EMA, Cytarabine 500mg/m2 CI days 1-3 & 8-10, Mitoxantrone 12 mg/m2 days 1-3 and Etoposide 200 mg/m2 CI days 8-10
G-CLAC, G-CSF, clofarabine, and high dose cytarabine
CR, Complete remission; NR, Non-responder

**Table S3. Gene and exon coverage of ThunderBolts Myeloid Panel (TBMP)**

| 49 Target Genes | Target Exons |
|---|---|
| ASXL1 | Full |
| BCOR | Full |
| BCORL1 | Full |
| BRAF | 11,12,13,14,15 |
| CALR | 9 |
| CBL | 8,9 |
| CBLB | 9,10 |
| CEBPA | Full |
| CSF3R | 14, 17 |
| DNMT3A | Full |
| ETV6 | Full |
| EZH2 | Full |
| FLT3 | 14,15,20 |
| GATA1 | 2 |
| GATA2 | 2,3,4,5,6 |
| GNAS | 8,9 |
| HRAS | 2,3,4 |
| IDH1 | 4 |
| IDH2 | 4 |
| JAK1 | 9,13 |
| JAK2 | 12,14 |
| JAK3 | 13 |
| KDM6A | Full |
| KIT | 8,9,10,11,13,17 |
| KMT2A/MLL-PTD | 1,3,5,6,7,8,27 |
| KRAS | 2,3,4 |
| MEK1 | 2,3 |
| MPL | 10 |
| MYD88 | 3,4,5 |
| NOTCH1 | 26,27,28,34 |
| NPM1 | 12 |
| NRAS | 2,3,4 |
| PHF6 | Full |
| PML | 3 |
| PTEN | 5,7 |
| PTPN11 | 3,13 |
| RAD21 | Full |
| RUNX1 | Full |
| SETBP1 | 4 |
| SF3B1 | 13,14,15,16 |
| SMC1A | Full |
| SMC3 | 10,13,19,23,25,28 |
| SRSF2 | 1 |
| STAG2 | Full |
| TET2 | Full |
| TP53 | Full |
| U2AF1 | 2,6 |
| WT1 | 7,9 |
| ZRSR2 | 2-5, 7-11 |
